# Spectral analysis of HIV seropositivity among migrant workers entering Kuwait

**DOI:** 10.1186/1471-2334-8-37

**Published:** 2008-03-25

**Authors:** Saeed Akhtar, Hameed GHH Mohammad

**Affiliations:** 1 Department of Community Medicine and Behavioural Sciences, Faculty of Medicine, Kuwait University, PO Box 24923, Safat 13110, Kuwait; 2 Ports and Borders Health Division, Ministry of Health, PO Box 32830, Rumaithiya 25410, Kuwait

## Abstract

**Background:**

There is paucity of published data on human immunodeficiency virus (HIV) seroprevalence among migrant workers entering Middle-East particularly Kuwait. We took advantage of the routine screening of migrants for HIV infection, upon arrival in Kuwait from the areas with high HIV prevalence, to 1) estimate the HIV seroprevalence among migrant workers entering Kuwait and to 2) ascertain if any significant time trend or changes had occurred in HIV seroprevalence among these migrants over the study period.

**Methods:**

The monthly aggregates of daily number of migrant workers tested and number of HIV seropositive were used to generate the monthly series of proportions of HIV seropositive (per 100,000) migrants over a period of 120 months from January 1, 1997 to December 31, 2006. We carried out spectral analysis of these time series data on monthly proportions (per 100,000) of HIV seropositive migrants.

**Results:**

Overall HIV seroprevalence (per 100,000) among the migrants was 21 (494/2328582) (95% CI: 19 -23), ranging from 11 (95% CI: 8 – 16) in 2003 to 31 (95% CI: 24 -41) in 1998. There was no discernable pattern in the year-specific proportions of HIV seropositive migrants up to 2003; in subsequent years there was a slight but consistent increase in the proportions of HIV seropositive migrants. However, the Mann-Kendall test showed non-significant (*P *= 0.741) trend in de-seasonalized data series of proportions of HIV seropositive migrants. The spectral density had a statistically significant (*P *= 0.03) peak located at a frequency (radians) 2.4, which corresponds to a regular cycle of three-month duration in this study. Auto-correlation function did not show any significant seasonality (correlation coefficient at lag 12 = – 0.025, *P *= 0.575).

**Conclusion:**

During the study period, overall a low HIV seroprevalence (0.021%) was recorded. Towards the end of the study, a slight but non-significant upward trend in the proportions of HIV seropositive migrants was recorded. A significant rhythmic cycle of three-month duration was observed in the proportions of HIV seropositive migrants. The underlying factors for a consistent upward trend towards the end of study period and for a significant quarterly cycle in the proportions of HIV seropositive migrants merit further investigations.

## Background

The human immunodeficiency virus (HIV) which emerged in the last quarter of twentieth century has spread worldwide in less than two decades [1]. Among various diversified factors, human migration in several parts of the world has played a significant role in HIV transmission [2]. Empirical evidence suggests that migrants and their host communities have increased vulnerability to HIV infection. Data from south-Asia and Africa suggest that mobility and migration separates people from their social support structures, creating social milieu in which they are more likely to engage in risk behaviours that are known to play a key role in spreading the HIV epidemic in other areas [3-7]. Therefore, migrants constitute a high risk group both for acquisition and a core group for HIV transmission [8]. Over the last decade, in most countries HIV/AIDS surveillance has been established by national AIDS programmes in collaboration with World Health Organization. The estimates from HIV surveillance can be used routinely in assessing the magnitude of the problem and in keeping intervention activities focused when estimates are available by sub-populations. These estimates also help in tracking the development of regional epidemics in the context of global situation [1].

Migrants from countries with a high prevalence of HIV/AIDS, notably from South Asia, South-east Asia and sub-Saharan Africa, bear a disproportionate and increasing share of infection with HIV throughout Western Europe [9]. However, there is paucity of published data on HIV seroprevalence among migrants from these regions entering Middle-East particularly Kuwait. Measuring the HIV seroprevalence among these migrants can be used as an additional surveillance method in Gulf countries with low or concentrated HIV prevalence. Here, we take advantage of the routine screening of migrant workers for HIV infection, upon arrival in Kuwait from areas with high HIV prevalence, to do the first large-scale quantification of the HIV infection status of this work population. Specifically, the cumulated serological data on HIV tests' results for these workers over the past ten years gave us an opportunity in this study not only to undertake 1) the estimation of HIV seroprevalence in this population of migrant workers, but also to 2) ascertain if any significant time trend or changes had occurred in the HIV seroprevalence among these migrants over the study period.

## Methods

### Setting and study population

Kuwait is a small oil-rich Gulf state having a total population of 2.5 million (Kuwaiti: 42%; Non-Kuwaiti 58%), with a gender ratio (male/female) of 1.04 at birth among nationals. Non-Kuwaiti constitutes about 80% of the labour force, and majority of them usually have a low educational attainment. Of the migrants, 46% are 20 to 44 year old and predominantly live as single, mainly because of their inability to fulfill a legal requirement of minimum wages to be able to bring their families [10]. In public sector, health-care system is made up of six administratively independent health-care sites; each comprises a general hospital, a health center, specialized clinics and dispensaries. Health services are free for all citizens and residents in Kuwait. The Middle East and North Africa region of UNAIDS continued to experience relatively low HIV/AIDS burden as the estimated number of new HIV infected cases duing 2007 were about 35000, which occurred mainly in men and in the urban areas [11]. In Kuwait, the number of known AIDS cases had been low (n = 106) from 1984 to 2004, nearly half of them were reported in adults (20–39 years) and 83% had heterosexual transmission [12].

### Data source

Monthly aggregates of the daily serological test results for HIV infection among migrants entered Kuwait between January 1, 1997 and December 31, 2006 were available for this study. These migrants predominantly came from India (31%), Bangladesh (14%), Sri-Lanka (14%), Egypt (12%), Indonesia (9%), Philippine (5%), Pakistan (5%) and 10% from other countries including those from African counties such as Tanzania, Mali, Gambia, Sudan [13,14]. Routine consensual medical examination procedures are conducted on these workers upon their arrival by the Ports & Borders Health Division of the Ministry of Health, Kuwait. For the diagnosis of HIV infection, serum samples are tested in Virology Laboratory of the Department of Public Health using commercially available 3rd generation enzyme-linked immunosorbent assay (ELISA) kits (Abbott). All the ELISA positive samples are further confirmed by Western blot analysis (Bio-Rad test) [15].

### Ethics

As noted above, on arrival in Kuwait, migrants are screened for various infections including HIV before issuance of residency permit. Verbal consent is solicited after fully informing each migrant about the purpose of serological screening. These procedures are performed according to a stated governmental policy. The study protocol was approved by the Ethics Review Committee of Faculty of Medicine, Kuwait University.

### Analytic approach

The monthly aggregates of daily number of migrant workers tested and number of HIV seropositive were used to generate the monthly series of proportions of HIV seropositive (per 100,000) migrants over a period of 120 months from January 1, 1997 to December 31, 2006. These monthly proportions (per 100,000) of HIV seropositive workers were used for all further analyses unless stated otherwise. We computed the year-specific proportions (95% confidence interval (CI)) of HIV seropositive migrants. The Mann-Kendall test (a non-parametric rank-based test) was conducted for assessing the significance of the trend in the de-seasonalized time series of proportions of HIV seropositive migrants. We carried out frequency domain (spectral) analysis of the de-seasonalized and de-trended time series data [16]. Spectral analysis detects the periodicity in time series by plotting the periodogram or spectral density of the series against period or frequency [17]. The power spectrum (frequency) distribution function was used to identify significant cycle in the proportions of HIV seropositive migrants. Significant deviations of the power spectrum from the trend of the spectrum represent the presence of cycle and regular or irregular (noise) patterns in the data. The Fisher's spectrum test was conducted to evaluate the null hypothesis that the series is strictly Gaussian white noise against the alternative hypothesis that the series contains an added deterministic periodic component of unspecified frequency. This test is designed to detect one major sinusoidal component buried in white noise [16]. The autocorrelation function was then used to measure correlation between observations at different time lags. A strong correlation between the observations at 12 time lags indicates a strong seasonality of the period [17].

## Results

**Figure 1:**
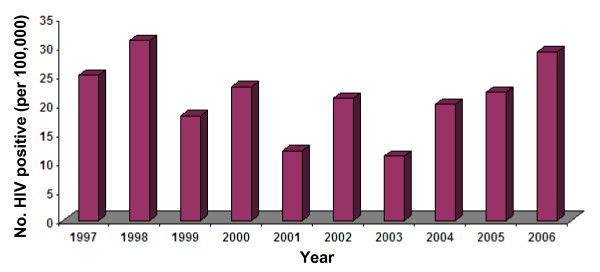
Year-specific HIV seroprevalence (per 100,000) among migrants entered Kuwait: 1997–2006.

**Figure 2:**
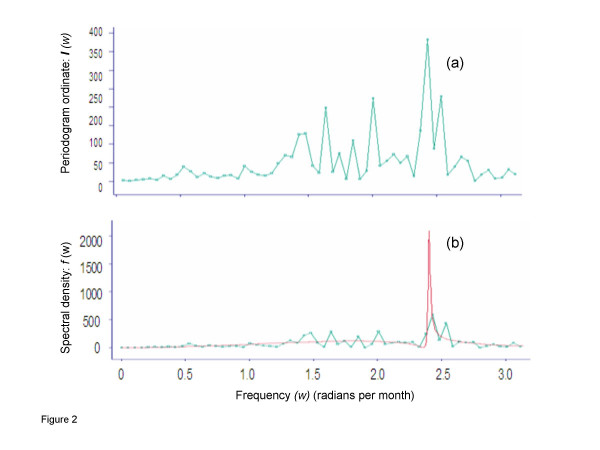
**Spectral analysis of monthly HIV seropositive migrants entered Kuwait: 1997–2006: (a) Periodogram; it describes the amplitude and spacing of any sinusoidal patterns in the data.** The value of the periodogram at a particular frequency (*ω*) is referred to as periodogram ordinate {I(*ω*)} as a function of *ω*; *ω *is measured as radians per month, which is convertible into portion of the cycle that is completed in one month). **(b) Power spectrum; detects significant signals (*i.e*. frequencies) buried in white-band noise.**

During the 120-month study period from January 1, 1997 to December 31, 2006, HIV serological test results for 2328582 migrant workers were included in this analysis. Overall HIV seroprevalence (per 100,000) among the migrants was 21 (494/2328582) (95% CI: 19 -23), (or 0.021%) with a range from 11 (95% CI: 8 – 16) in 2003 to 31 (95% CI: 24 -41) in 1998. There was no discernable pattern in year-specific proportions of HIV seropositive migrants up to 2003. However, in subsequent years there was a slight but consistent increase in the proportions of HIV seropositive migrants (Figure 1, Table 1). The Mann-Kendall test showed non-significant (*P *= 0.741) trend in the de-seasonalized data series of proportions of HIV seropositive migrants. The estimated periodogram and power spectrum of data series revealed a statistically significant (*P *= 0.03) peak in spectral density located at a frequency (radians) 2.4 (Figure 2). In this study, this frequency value corresponds to a regular cycle of three-month duration. The auto-correlation function did not show any significant seasonality in the monthly proportions of HIV seropositive migrants (correlation coefficient at lag 12 = – 0.025, *P *= 0.575).

**Table 1:** Year-specific distribution of proportions of HIV seropositive migrant workers entering Kuwait: 1997–2006

Year	Total tested	No. positive	No. Positive (per 100, 000)	95% confidence interval
1997	161682	40	25	18 – 34
1998	163326	51	31	24 – 41
1999	177129	31	18	12 – 25
2000	130984	30	23	16 – 33
2001	178472	21	12	7 – 18
2002	221566	46	21	16 – 28
2003	254608	29	11	8 – 16
2004	327216	66	20	16 – 26
2005	356983	77	22	17 – 27
2006	356616	103	29	24 – 35

Overall prevalence (per 100,000) of HIV seropositive migrant workers = 21 (494/2328582); (95% Cl: 19 – 23) (or 0.021%)

## Discussion

We used the frequency domain analysis to estimate the spectrum over the whole range of frequencies. These techniques are widely used in the fields of electrical engineering, physics, meteorology, marine science [16], and epidemiology [18,19]. Spectral analysis has two advantages which make it well suited for epidemiologic applications. First, it permits the time series data to be disaggregated into several variables represented by sine waves, each with its own frequency, phase and amplitude. Second, in this sinusoidal form, the time series data is well suited for linear filtering, which helps in evaluation of more subtle patterns in the data [16]. The periodogram examines the frequency of different oscillations of the observed time-series and specifically designed for detection of periodic patterns in a background of white noise. The purpose of spectral estimation is to describe the distribution (over frequency) of the power contained in a time-series, based on a finite data set. Estimation of power spectrum includes detection of significant signals buried in white-band noise [17].

Population level studies for assessment of magnitude of HIV infection suffer from potential biases, which are likely to hamper the validity and reliability of results [20]. Therefore, population-based registries are progressively becoming the best tool for HIV surveillance in Europe [21]. However, this type of registration of HIV infected patients yet to be implemented in less developed countries. Nevertheless, one of the best ways to monitor the evolution of HIV infection is the measurement of HIV incidence and serial HIV prevalences among the high risk groups over long periods of times. The immigrants constitute one of such high risk groups for acquisition and transmission of infectious diseases because of their risk behaviours [22]. The Middle East in general and Kuwait in particular has a major influx of immigrants from countries with high HIV prevalence and the paucity of published data on the magnitude of HIV infection among these migrants was a main motivation for this analysis.

During the entire study period of 120 months, HIV seroprevalence (per 100,000) among the migrants was 21 (95% CI: 19 – 23) or 0.021%, which is substantially lower than 5.2% reported among immigrants in Europe [23]. Relatively low HIV seroprevalence among these migrants during the study period may be reflecting a phase of HIV epidemic in the countries of their origins [24]. Also, in this study, no significant trend in the year-specific proportions of HIV seropositive migrants was noted up to 2003. But in the remaining part of the study period, a slight but consistent increase in the proportions of HIV seropositive migrants was recorded. This observed increase in the proportions of HIV seropositive migrants may be mimicking the current status of HIV epidemic, which is evidently flaring-up at present [22]. However, the factors associated with the slight increase in HIV seropositivity among these migrants towards the end of study period merit further investigations.

This study provides descriptive data on the periodicity in HIV seropositivity among migrants entering Kuwait predominantly from South-Asian countries, wherein, HIV epidemic is flaring-up recently [22]. In this study, evaluation of probable factors associated with the observed periodicity was not contemplated. However, rhythmic patterns in HIV seropositivity among these migrants provided us the opportunity to point out some considerations as to their origins and implications. The proportions of HIV seropositive migrants in this study have a significant cycle of 3-month duration. We are unaware of any previously published literature that has demonstrated analogous cyclic pattern in HIV seropositivity among migrating populations. However, a study from West Africa has implicated that mobility appeared to be a key factor in HIV spread, not only because population movements enable the virus to disseminate but also because of particular risk behaviours of those who are mobile [25]. The biological basis for quarterly rhythm in the proportions of HIV seropositive migrants in our study is unclear. However, it may be reflecting an underlying migrants' recruitment pattern from a particular region and/or from a particular segment of the population of the countries of their origins. We did not have the access to the data on migrants' demographic variables and on their exact recruitment locations within the countries of their origins to substantiate this contention. However, a previous study has shown that among immigrants in Europe, the frequency of specific infectious diseases including infection with HIV was strongly associated with the areas of their origins [23]. Furthermore, spatial heterogeneity in HIV incidence, which depends on several social, demographic and behavioural factors both at the population and the individual level, is well known in less developed Asian and African countries [24,26]. Nevertheless, further aetiological investigations of the basis for this observed quarterly rhythmic cycle in the proportions of HIV seropositive migrants are needed.

Limitations of this analysis included lack of demographic data on the migrants and their spatial distributions within the countries of their origins, which precluded the analysis to take into account potential differentials in HIV seroprevalence.

## Conclusion

In conclusion, this study showed overall a low HIV seroprevalence (0.021%) among the migrants entering Kuwait. Towards the end of the study period, however, there was a slight but non-significant upward trend in the proportions of HIV seropositive migrants. A significant rhythmic cycle of three-month duration was observed in the proportions of HIV seropositive migrants. The underlying factors for upward trend towards the end of study period and the significant quarterly cycle in HIV seropositivity among migrants merit further investigations.

## Competing interests

The author(s) declare that they have no competing interests.

## Authors' contributions

SA conceived, design, analyzed, interpreted the data and drafted the manuscript. HGHHM supervised data collection and reviewed the manuscript. Both the authors have read and approved the final manuscript.

## Pre-publication history

The pre-publication history for this paper can be accessed here:


